# PUR-GEN: A web server for automated generation of polyurethane fragment libraries

**DOI:** 10.1016/j.csbj.2024.12.004

**Published:** 2024-12-12

**Authors:** Katarzyna Szleper, Mateusz Cebula, Oksana Kovalenko, Artur Góra, Agata Raczyńska

**Affiliations:** a Tunneling Group, Biotechnology Centre, Silesian University of Technology, Bolesława Krzywoustego 8, Gliwice 44-100, Poland; b Faculty of Automatic Control, Electronics and Computer Science, Silesian University of Technology, Gliwice, Poland; c Department of Organic Chemistry, Bioorganic Chemistry and Biotechnology, Faculty of Chemistry, Silesian University of Technology, Gliwice, Poland; d Toulouse Biotechnology Institute, TBI, Université de Toulouse, CNRS, INRAE, INSA, 135 avenue de Rangueil, Toulouse F-31077 Cedex 04, France

**Keywords:** Polyurethanes, Isocyanates, Polyols, Biodegradation, Cutinases, Urethanases

## Abstract

The biodegradation of synthetic polymers offers a promising solution for sustainable plastic recycling. Polyurethanes (PUR) stand out among these polymers due to their susceptibility to enzymatic hydrolysis. However, the intricate 3D structures formed by PUR chains present challenges for biodegradation studies, both computational and experimental. To facilitate *in silico* research, we introduce PUR-GEN, a web server tailored for the automated generation of PUR fragment libraries. PUR-GEN allows users to input isocyanate and alcohol structural units, facilitating the creation of combinatorial oligomer libraries enriched with conformers and compound property tables. PUR-GEN can serve as a valuable tool for designing PUR fragments to mimic PUR structure interactions with proteins, as well as characterising simplistic PUR models. To illustrate an application of the web server, we present a case study on selected four cutinases and three urethanases with experimentally confirmed PUR-degrading activity or ability to hydrolyse carbamates. The use of PUR-GEN in molecular docking of 414 generated oligomers provides an example of the pipeline for initiation of the PUR degrading enzymes discovery.

## Highlights

• PUR-GEN: Automated PUR fragment generation web server.
• Facilitates diverse PUR structure exploration.
• Enables rapid preparation of ligand structures.
• Enhances enzyme scaffold identification for PUR degradation.
• Valuable resource for sustainable polymer management.

## Graphical Abstract

**Figure:**
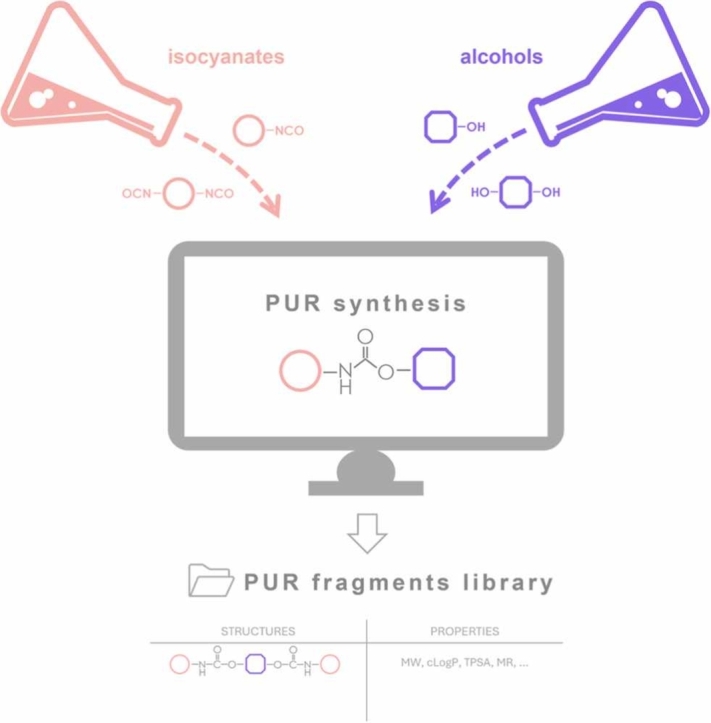

## 1 Introduction

Biodegradation of synthetic polymers is considered as an alternative approach to plastics recycling and upcycling. Compared to these methods, biodegradation demonstrates lower energy requirements throughout the supply chain and reduced greenhouse gas emissions [1], [2], [3], [4], [5], [6]. Polyurethanes (PUR) are the sixth most produced polymer family worldwide and are of particular interest in this context, as they contain urethane groups in their chains that are susceptible to enzymatic hydrolysis. Moreover, polyester PURs also feature easily degradable ester bonds, making them potentially suitable for biological recycling [7], [8], [9], [10].

**Fig. 1:**
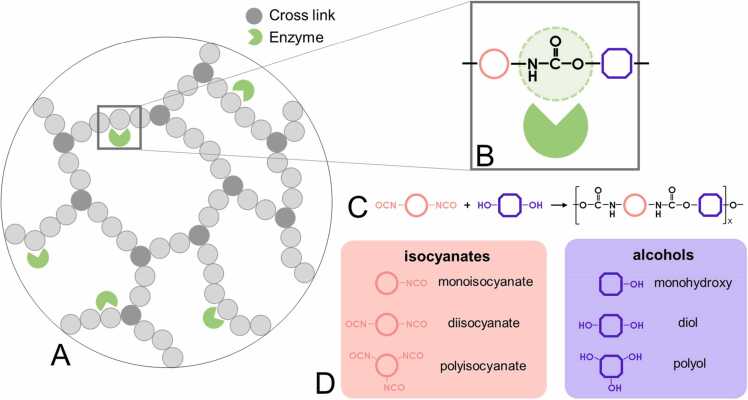
Structure, synthesis and structural units of PURs. A) Schematic representation of a cross-linked PUR (thermoset) together with enzymes binding to its structure. B) Simplified representation of the enzyme and PUR fragment system. PUR fragment contains a urethane bond and two comonomers. C) Common route for the synthesis of PUR. D) Types of comonomers (isocyanates and alcohols) used for synthesis of PUR based on the number of their functionalities.

PURs are typically synthesised through the reaction between isocyanates (N = C = O) and alcohols (-OH), resulting in polymer formation with these comonomers connected by urethane bonds (Fig. 1C) [11], [12]. Isocyanate and alcohol units serve as the fundamental structural units of PUR, with the incorporation of chain extenders and cross-linkers allowing the production of PUR structures with varying mechanical properties. Structural units with a single functional group (e.g. monoisocyanates and monohydroxy alcohols) lead to the formation of PUR fragments (oligomers) and carbamates [13], [14], [15], [16]. By synthesis of PUR from structural units containing two functional groups (e.g. diisocyanates and diols), long linear polymers called thermoplastics are formed. Cross-linking occurs when compounds containing more than two functional groups are introduced (e.g. polyisocyanates and polyols), resulting in the synthesis of branched, three-dimensional PURs known as thermosets (Fig. 1D). Additionally, polyols may contain other functional groups such as ester or ether groups in the chain backbone.

The complex 3D structures formed by PUR chains, often enhanced by cross-linking, present significant challenges for biodegradation studies (Fig. 1A). Commercially available PUR structures are frequently proprietary, with limited information regarding their chemical composition and molecular weight. Consequently, PURs represent a challenging experimental and computational target for biodegradation research [17], [18], [19], [20], [21], [22], [23], [24], [25], [26], [27], [28]. To address this, researchers sometimes synthesise PURs themselves, enabling their thorough characterisation [6], [29], [30], [31], [32], [33], [34], [35], [36], however, the lack of a systematic and broader view of these issues is readily apparent.

In PUR biodegradation studies, computational methods can be used to identify enzyme candidates for subsequent modification and experimental validation. These methods necessitate accurate modelling of PUR substrates, which involves generating a diverse set of compounds that represent the variability of PUR. Modelling entire 3D PUR structures presents considerable challenges, leading researchers to focus on local interactions between low molecular weight PUR fragments and enzymes (Fig. 1B). Consequently, computational and experimental studies are often limited to a few representative monomers or PUR fragments [15], [37], [38], [39], [40], [41]. Such an approach, however, may overlook the highly heterogeneous and diverse nature of commercially used PURs.

Another significant challenge in the experimental analysis and validation of PUR degradation products is the lack of standardised approaches [42]. Addressing this gap requires methods that enable quantitative verification of PUR degradation level, as well as model substrates with well-defined characteristics covering the full spectrum of degradation products. These advancements are essential for validating computational results and refining computational methods, ultimately leading to more accurate predictions in PUR biodegradation studies.

To address these challenges and facilitate PUR fragments modelling, we introduce PUR-GEN, a web server (https://pur-gen.polsl.pl) designed for the automated generation of PUR fragments libraries. PUR-GEN allows users to input selected isocyanate and alcohol structural units or choose from a predefined collection, generating a library of oligomers containing urethane bonds. Users can specify the desired number of repeating units and terminal groups for the generated PUR fragments. The output library includes oligomers in structural file formats (.mol and.mol2), enriched with conformers, 2D visualisations and compound property tables.

In this work, we present PUR-GEN as a valuable tool for generation of the library of PUR fragments with easy access to their structures and characteristics. We anticipate that PUR-GEN generated libraries of compounds will facilitate molecular docking studies to investigate interactions between PURs and proteins, facilitating enzymes engineering, as well as aid in the design of PUR fragments for experimental synthesis and high-throughput study. Additionally, we provide an illustrative use case example of PUR-GEN's application, analysing molecular docking results of 414 oligomers to four cutinases and three urethanases with experimentally confirmed PUR-degrading activity or ability to cleave urethane bonds in (di)carbamates, providing fast insight into their individual properties of fundamental importance for their further engineering. While the example presented demonstrates a sample pipeline, it does not exhaust the potential applications of webserver-generated fragments for other types of research.

## 2 PUR-GEN

The PUR-GEN (https://www.pur-gen.polsl.pl) web application represents a novel chemoinformatic tool tailored for the systematic generation of fragments of PUR compounds, either individually or in libraries. The tool uses open-source Python libraries such as RDKit [43] and Pybel [44], and special languages SMILES [45] and SMARTS, to encode chemical structures and reactions.

**Fig. 2:**
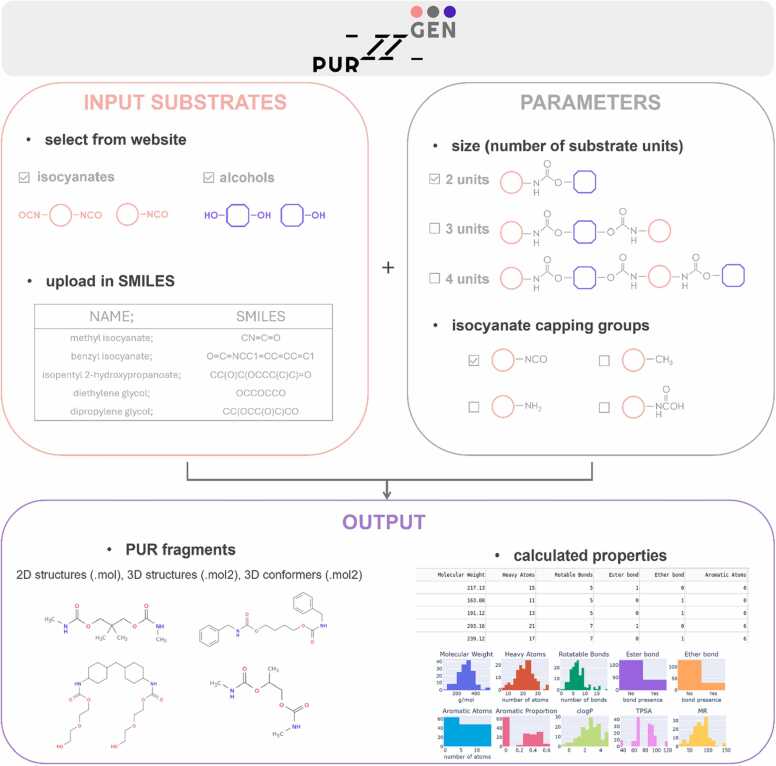
Schematic representation of PUR-GEN application workflow. PUR fragments can be generated from input structural units (isocyanates and alcohols) provided within the webserver or from other compounds uploaded by the user in SMILES format. Length of generated PUR fragment can be specified by the number of structural units (2, 3 or 4). Free isocyanate moieties in obtained fragments can be capped with amine, methyl, carbamate capping group. The output from the PUR-GEN web application includes structural files (.mol and.mol2), enriched with their conformers, their 2D visualisation and a table containing their properties.

The primary interface of PUR-GEN integrates essential navigation elements, including a direct link to an instructional tutorial ("How to use") and access to the calculation preparation page ("Run PUR-GEN"). Within this interface, as input, the user can select pre-existing structures or upload their own isocyanates and alcohols in SMILES format. These compounds serve as foundational comonomers in the generated PUR fragments (Fig. 2). The webserver presently hosts a collection of 10 isocyanate structures that are often used in industrial applications (Supplementary Fig. S1). The tool also provides a range of 16 hydroxyl compounds, which enables introduction of ester or ether bonds into the resulting PUR fragments, closely mimicking the composition of commercially utilised polyether and polyester polyols (Supplementary Fig. S2). Our webserver is designed to provide compound libraries that resemble polymer chains for further modelling studies, rather than the entire 3D structure of PURs. Therefore, crosslinking structural units with more than two functional groups are excluded from the webserver to maintain synthesis feasibility and user clarity.

**Fig. 3:**
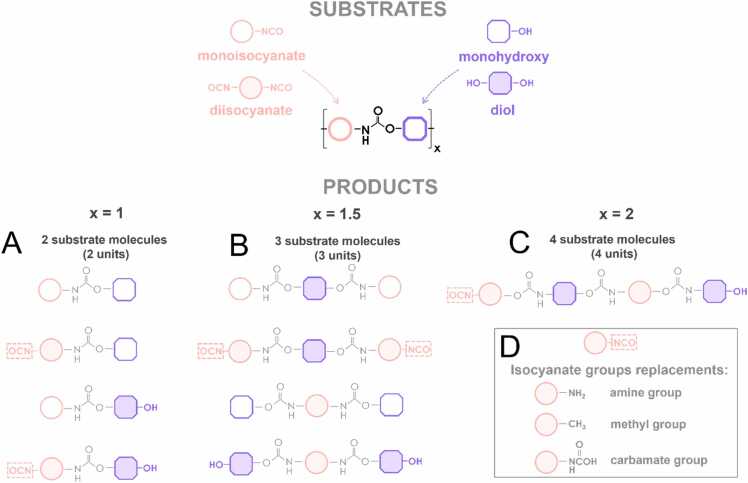
Possible combinations of mono- and difunctional isocyanates and alcohols to generate PUR fragments as A) 2 units, B) 3 units, C) 4 units. D) Reactive isocyanate moieties can be replaced with another neutral capping group, one of amine (-NH2), methyl (-CH3) or carbamate (-NC(=O)OH).

Users are granted the flexibility to specify the desired length of the PUR fragments they aim to generate. The minimum "2 units" fragments contain one molecule of each comonomer connected by a urethane bond (Fig. 3A). Then, "3 units" consist of three structural units: two molecules of one comonomer and one of the other. The structural unit in the middle must have two functional groups (-NCO or -OH) (Fig. 3B). The longest one, "4 units" consists of four structural units; two molecules of each comonomer (Fig. 3C). The synthesis of such a fragment requires the use of structural units with two functional groups each.

Depending on the number of functional groups in the structural units employed, the resulting PUR fragments may contain reactive isocyanate moieties (-N = C = O). The user has the option of replacing these groups with another neutral capping group, one of amine (-NH2), methyl (-CH3) or carbamate (-NC(=O)OH) (Fig. 3D). Such capping allows to reduce the partial charge on atoms, which originates from the discontinuity of the modelled polymer chain and facilitates downstream molecular docking experiments.

Once the user specifies the input structures and parameters—such as fragment lengths and capping groups—the PUR-GEN web application systematically combines the selected isocyanates and alcohols to generate PUR fragments. Using functions in the RDKit [43] library and reaction patterns encoded in SMARTS, it merges -N = C = O groups in isocyanates with -OH groups in alcohols to form urethane bonds. Additionally, PUR-GEN offers a comprehensive analysis of the resulting PUR fragments, encompassing essential molecular properties. These properties include molecular weight, heavy atom count, number of rotatable bonds, presence of ester and ether bonds, count of aromatic atoms, aromatic proportion (ratio of aromatic atoms to heavy atoms), Crippen-Wildman partition coefficient (cLogP), Crippen-Wildman molar refraction (MR) [46] and topological polar surface area (TPSA) [47]. Users can conveniently access and download these calculated properties in tabular format (.csv) for further analysis. Furthermore, PUR-GEN allows users to download the generated structures in.mol and.mol2 file formats, facilitating compatibility with various molecular modelling and visualisation software. For enhanced structural exploration, the application offers the option to generate up to 20 conformers utilising Pybel library [44] and Confab [48] method. However, due to limitations inherent to this method, larger or more complex compounds generated in PUR-GEN may yield fewer conformers, sometimes as few as one. Generating conformers for large ligands remains challenging, primarily due to the combinatorial explosion in conformational space as the number of rotatable bonds increases. These challenges stem from limitations in the Pybel library and associated Confab algorithm, rather than being specific to PUR-GEN. Notably, some molecular docking tools, such as AutoDock Vina [49], [50], employed in the use case presented in this manuscript, allow for ligand flexibility, generating docked poses in various conformations. Conversely, in molecular docking methods reliant on rigid ligand docking, a fragment-based docking approach may be an effective alternative.

## 3 Use case example – molecular docking analysis

In this chapter, we show an example of a user study in which we initially compare the potential of selected enzymes for their further employment in engineering towards effective PUR degradation. A library of PUR fragments curated from PUR-GEN webserver was docked to enzyme representatives of two groups: cutinases (EC 3.1.1.74) and urethanases (EC 3.5.1.75). Since, adequate substrate binding is a prerequisite (but not sufficient condition) for the initiation of biocatalysis, it can be used to preselect best enzymes candidates for further redesign.

### 3.1 PUR library characterisation

The set of 414 compounds employed in this study was systematically generated by exhaustively exploring all possible combinations of structural units (10 isocyanates and 16 polyols) available in the in-house version of PUR-GEN. The resulting library of PUR ligands exhibits diverse structural properties, as depicted in Supplementary Figure S3. Notably, the composition of the ligands varies based on the number of comonomers used in their synthesis. Specifically, ligands containing one urethane bond (referred to as 2 units) constitute 39% of the compounds, while those featuring two (3 units) and three (4 units) urethane bonds represent 48% and 13% of the library, respectively. The length of designed structures exhibits large variation, with the number of heavy atoms ranging from 6 to 72, and molecular weights spanning from 89 to 1027 unified atomic mass units (u).

Further analysis reveals variations in aromaticity among the ligands, contingent upon the isocyanate structural units employed. Ligands within the library display a distribution wherein 39% contain no aromatic atoms, 19% feature one aromatic ring, 32% comprise two aromatic rings, and 10% exhibit four aromatic rings. Additionally, depending on the hydroxyl compound utilised, ligands may contain additional functional groups such as ester bonds (17%) or ether bonds (26%). The calculated cLogP for the compounds ranges from –0.7–9.8, indicative of a spectrum ranging from neutral to hydrophobic ligands. This diversity in structural and physicochemical properties of in house implemented building blocks, underscores the potential of the generated PUR fragment library to serve as a valuable resource for exploring interactions with target enzymes and elucidating structure-activity relationships. It should be noted that users have a full freedom to extend or limit building blocks library to optimally mimic investigated PUR structure.

### 3.2 Enzymes selected for the study

Cutinases are enzymes capable of hydrolysing cutin, a complex biopolymer that forms plant cuticle. They are classified as serine hydrolases and share an alpha/beta fold. However, notable differences exist between fungal and bacterial cutinases: fungal cutinases are generally smaller in size (∼20 kDa) compared to their bacterial counterparts (∼28 kDa). Bacterial cutinases typically feature nine beta-sheets surrounded by alpha helices [51], while fungal cutinases, being smaller, exhibit only five beta sheets [52] (Supplementary Fig. S4). In this study, we selected four cutinases: two from fungal sources—CpCut1, derived from *Cladosporium sp.* P7 [53] and HiC from *Humicola insolens* [30]— and two from bacterial sources—LCC from leaf compost metagenome [7] and TfCut2 from *Thermobifida fusca* [7]. The analysed cutinases cleave ester bonds in polyester PUR [54], [55], [56], [57], [58], and are potentially capable of urethane bond hydrolysis [30], [59], [60].

The second group of enzymes selected are urethanases, which catalyse the cleavage of urethane bonds to release amines, alcohols, and carbon dioxide. The amino acid sequences of urethanases share the conserved GGSS(S/G)GS motif, a signature of the amidase family [61], [62], [63] (Supplementary Fig. S5). We selected 3 enzymes as representatives. UMG-SP-1 and UMG-SP-2 were identified from a metagenomic library created from DNA isolated from an enriched site containing perennial PUR waste. Their urethanase activity was confirmed on various dicarbamates, which are structurally similar to PUR fragments [64]. Recently, UMG-SP-1 was also shown to depolymerise polyester PUR and a selected polyamide (PA) through the cleavage of urethane and amide bonds, respectively [61]. The third urethanase, AmdA, from *Agrobacterium tumefaciens* d3, has been confirmed to hydrolyse ethyl carbamate [62], [65].

Cutinases and urethanases share a similar mechanism, performing hydrolysis *via* nucleophilic attack by the catalytic serine on the carbonyl carbon of the cleaved bond [66], [67]. Both enzyme groups also have an oxyanion hole, critical for stabilising the tetrahedral intermediate formed during the acylation step. However, location and access to the active site of cutinases and urethanases shows remarkable differences. Cutinases have exposed binding sites on the enzyme surface, comprising numerous hydrophobic residues, in contrast, urethanases have a buried active site with tunnels and pockets inside their core [67]. Cutinases possess a classical Ser-His-Asp [51] catalytic triad, whereas urethanases have a Ser-(cis)Ser-Lys catalytic triad [61].

### 3.3 Docking results

**Fig. 4:**
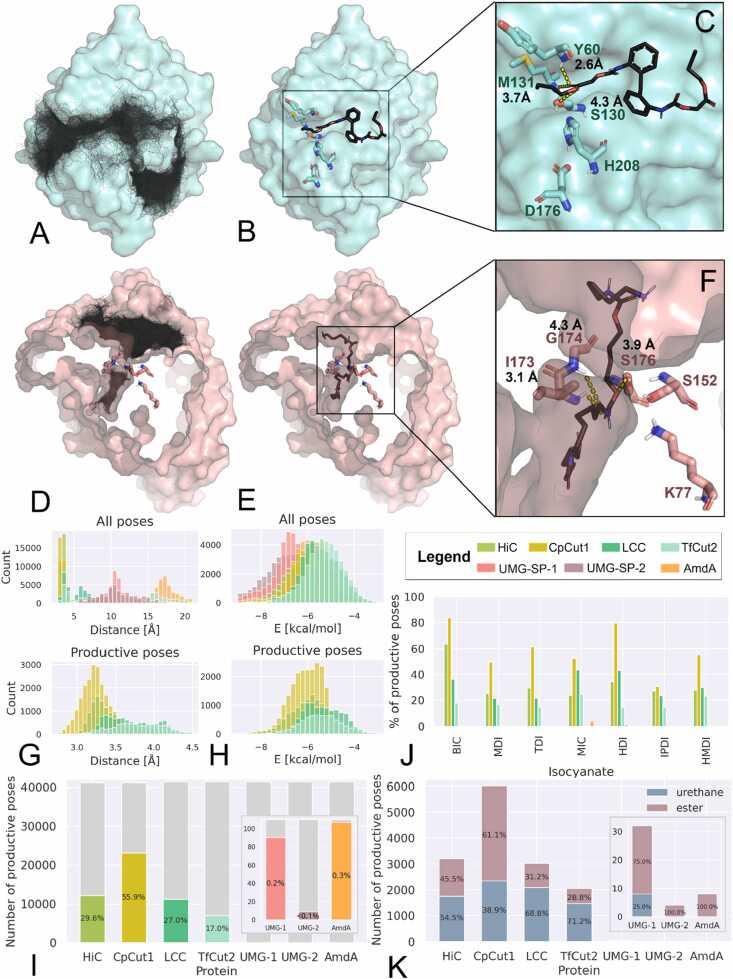
Results of docking studies for a generated library of 414 PUR fragments with four cutinases: HiC, CpCut1, LCC, TfCut2; and a urethanases UMG-SP-1. A) TfCut2 structure (cyan surface) with ten poses of all docked ligands from one repetition shown as transparent black lines (10 poses for each of the 414 ligands in one of ten repetitions). The transparent lines represent ∼4140 poses of 414 ligands, creating 'clusters' that indicate regions where ligands were frequently docked. B) TfCut2 structure (cyan surface) with catalytic triad and oxyanion residues shown as sticks. A chosen ligand (trimer_25 generated from 2,2-MDI and isopentyl 2-hydroxypropanoate) is displayed in a productive pose (black sticks). C) Productive pose of the selected ligand. TfCut2 structure (cyan surface) with catalytic triad and oxyanion residues shown as sticks. The key interactions are highlighted: (1) the distance between the catalytic serine (Ser130) oxygen and the carbonyl carbon of the cleaved ester bond in the ligand (yellow dashed line, 4.0 Å) and (2) the distances between the backbone hydrogen atoms of the oxyanion residues (Tyr60 and Met131) and the carbonyl oxygen of the cleaved ester bond in the ligand (yellow dashed lines, 2.6 Å and 3.7 Å, respectively). D) UMG-SP-1 structure (salmon surface) with ten poses of all docked ligands from one repetition shown as transparent black lines (10 poses for each of the 414 ligands in one of ten repetitions). The enzyme structure was sliced to show docked poses of the ligands inside the macromolecule’s core. The transparent lines represent ∼4140 poses of 414 ligands, creating 'clusters' that indicate regions where ligands were frequently docked. E) UMG-SP-1 structure (salmon surface) with catalytic triad and oxyanion residues shown as sticks. A chosen ligand (tetramer_23 generated from HDI and 1,4-butanediol) is displayed in a productive pose (black sticks). F) Productive pose of the selected ligand. UMG-SP-1 structure (salmon surface) with catalytic triad and oxyanion residues shown as sticks. The key interactions are highlighted: (1) the distance between the catalytic serine (Ser176) oxygen and the carbonyl carbon of the cleaved urethane bond in the ligand (yellow dashed line, 3.9 Å) and (2) the distances between the backbone hydrogen atoms of the oxyanion residues (Ile173 and Gly174) and the carbonyl oxygen of the cleaved urethane bond in the ligand (yellow dashed lines, 3.1 Å and 4.3 Å, respectively).G) Distribution of distances between the catalytic serine oxygen of each enzyme and the carbonyl carbon of the closest hydrolysable bond (urethane or ester) of the docked ligands. H) Distribution of binding affinity energies obtained for each enzyme. I) Percentage of productive and non-productive poses obtained for all ligands. J) Percentage of productive poses for each enzyme, categorised by the type of isocyanate used in ligand synthesis: ‘BIC’ (benzyl isocyanate), ‘MDI’ (4,4′-MDI, 2,4′-MDI, and 2,2′-MDI), ‘TDI’ (2,4′-TDI and 2,6′-TDI), ‘MIC’ (methyl isocyanate), ‘HDI’ (hexamethylene diisocyanate), ‘IPDI’ (isophorone diisocyanate), and ‘HMDI’ (4,4′-HMDI). K) Comparison of the number of productive orientations of urethane bonds versus ester bonds (for polyester PUR fragments containing both urethane and ester bonds).

In this study, we tested the potential substrate recognition pattern of preselected enzymes using library of oligomers prepared with the PUR-GEN webserver. We docked all 414 PUR fragments generated by PUR-GEN to four selected cutinases and three urethanases, using AutoDock Vina [49], [50]. For each protein, a total of approximately 41,400 docked poses were obtained (414 ligands x 10 repetitions x ∼10 docking poses) (Fig. 4A, 4D). These poses were analysed in terms of their position, orientation, and binding affinity, with a precise evaluation of the type of potentially attacked bond and the possibility of the substrate stabilisation by oxyanion hole. As an outcome, we provide information about the binding preferences of selected enzymes and compare the obtained data with enzyme activity reported in the literature.

The binding preferences were examined by evaluating ratio between productive and nonproductive poses. To determine whether a docked pose is bound in a potentially productive manner for nucleophilic attack, we used two key indicators: (1) the distance between the catalytic serine and the scissile bond, and (2) the distances between atoms involved in stabilising the oxyanion hole. The first indicator was defined as the distance between the oxygen atom of the catalytic serine in the enzyme and the carbonyl carbon of the nearest cleavable bond (urethane or ester) in the docked ligand pose. The second set of distances was measured between the backbone NH groups of oxyanion residues and carbonyl oxygen of the cleavable bond (Fig. 4C, F). We refer to such poses as near-attack configurations (NACs) or productive poses. Due to the limitations of the rigid receptor molecular docking method used in this study, we set an arbitrary threshold of 4.5 Å for all key distances. The percentage of poses that were deemed productive for each enzyme is shown in Fig. 4I, whereas the number of productive poses obtained for each ligand are shown in Supplementary Figs. S6-S8.

The results revealed that, for HiC, CpCut1, LCC and TfCut2 approximately 30%, 56%, 27%, and 17% of all docked poses were deemed productive, respectively (Fig. 4I). Notably, CpCut1 exhibited the highest percentage of productive poses, nearly double that of the next highest enzyme (HiC). This finding aligns with experimental reports highlighting the superior performance of CpCut1 compared to HiC, LCC, and TfCut2 [53], underscoring the significance of our observations in the context of established data. For the urethanases UMG-SP-1, UMG-SP-2 and AmdA, very few poses were deemed productive (0.2%, <0.1% and 0.3%, respectively). These results align with the observed accessibility of the active sites in both groups of enzymes. Cutinases, with their surface-exposed active sites, can accommodate a wide variety of compounds, suggesting their promiscuity. In contrast, the active sites of the tested urethanases, positioned at the end of a narrow tunnel, indicate a high specificity for ligands, most likely hydrolysing the terminal mers of PUR chains. The differences in the number of productive poses found for UMG-SP-1, UMG-SP-2, and AmdA are too small to indicate a significant distinction between the enzymes, especially considering that no enhanced sampling of protein conformations was performed. However, these results could provide indication of type of PUR polymer for activity testing. According to literature, UMG-SP-1 possess ability to depolymerise polyester PUR and PA chains *via* urethanolytic and amidolytic cleavage [61], and therefore might be better adapted to bind bulky PUR in comparison with UMG-SP-2.

The histograms depicting the distance (1) between the catalytic serine oxygen atom and the carbonyl carbon of the nearest hydrolysable bond (Fig. 4G) illustrate that for bacterial cutinases (LCC and TfCut2) and urethanases, many poses were docked either in unproductive configurations or even outside the active site cavity (distance for nucleophilic attack > 10 Å, Fig. 4A,D,G), indicating nonspecific binding of some poses.

When comparing the binding affinity results (Fig. 4H), the ligand poses docked to fungal cutinases exhibited lower energies than those docked to bacterial cutinases, for both productive and nonproductive poses. For urethanases, although a negligible number of poses were deemed productive (Fig. 4I), the calculated binding energies for the ligand poses were lower than those for the cutinases (Fig. 4H). The lower binding energies obtained for urethanases are associated to tight binding of the substrate in the narrow pocket. Urethanases have a buried active site and a restricted volume in the binding pocket, limiting their ability to accommodate bulky ligands (Fig. 4D-F). In UMG-SP-1, a bottleneck near catalytic S176 hinders the unrestricted binding of ligands in a productive pose (Fig. 4F). Consequently, many ligands were bound in the binding pocket within the protein core, but not in a pose where the carbonyl carbon of the cleaved group is at an appropriate distance for nucleophilic attack by S176.

Given that 17% of the generated oligomers are based on ester alcohols, which means that these ligands contain both urethane and ester bonds in their backbone, we analysed the preferential positioning of these bonds within the active site of each enzyme. Specifically, we investigated which of these bonds — urethane or ester — are in proximity to the catalytic serine, defined by the NAC distance, within the active site of the enzymes. The results revealed that, for HiC, CpCut1, LCC and TfCut2, respectively, approximately 55%, 39%, 69%, and 71% of productive poses of polyester PUR ligands had urethane bonds in the NAC configuration (Fig. 4K). Notably, while no urethanase activity has been reported for bacterial cutinases, urethane bond binding appears to be preferred over ester bonds (Fig. 4K). Conversely, for urethanases, ester bond binding seems to be preferred. This preference may arise from the higher steric hindrance of urethane bonds compared to ester bonds, which could be less compatible with the relatively tight binding pocket of urethanases. However, substrate preference in this case may not be due to restricted binding affinity but, as one study suggests [68], due to the enzyme's ability to stabilise the tetrahedral intermediate formed after the nucleophilic attack of the catalytic serine on the carbonyl carbon in both ester and urethane bonds. In a previous study, HiC’s activity was even shifted from esterolytic to amidolytic with a single amino acid substitution near the active site [69].

PURs are commonly classified according to the polyol type (ester or ether) and/or the type of isocyanate they are derived from, with PUR stability varying notably based on the isocyanate type [70]. Our generated PUR library includes a diverse range of isocyanates prevalent in industrial applications. This encompasses both aromatic structural units like benzyl isocyanate (BIC), 4,4′-methylenediphenyl diisocyanate (MDI), 2,4′-MDI, 2,2′-MDI, 2,4′-toluene diisocyanate (TDI), and 2,6′-TDI, as well as aliphatic moieties like methyl isocyanate (MIC), hexamethylene diisocyanate (HDI), isophorone diisocyanate (IPDI), and 4,4′-methylenedicyclohexyl diisocyanate (HMDI) (Supplementary Fig. S1). We divided the ligands into seven groups based on the type of structural isocyanate unit that each oligomer is based on. Fungal and bacterial cutinases were able to bind various isocyanate-based PURs in productive poses. However, UMG-SP-1 was mainly able to bind aliphatic HDI-based PURs, whereas AmdA – the smallest MIC-based PURs (Fig. J). Such results may suggest the types of enzymes that could serve as starting points for engineering aimed at degrading specific polyurethane polymers. Such results may suggest the types of enzymes that could serve as starting points for engineering aimed at degrading specific polyurethane polymers.

In summary, this molecular docking study characterised substrate binding preferences of selected representatives of two reported in literature PUR-degrading enzyme groups: cutinases and urethanases. The results indicate that cutinases, with their surface-exposed active sites, exhibit a high degree of promiscuity, as evidenced by the numerous productive ligand-binding poses. In contrast, urethanases have a buried active site that, while potentially facilitating the hydrolysis of more recalcitrant urethane bonds, can limit their ability to accommodate large PUR substrates or may necessitate conformational changes of the enzymes.

These findings suggest that incorporating representatives from both cutinases and urethanases into a polyester PUR biodegradation pipeline could facilitate concerted enzyme action, as was previously reported [71], [72], [73], [74]. Cutinases, capable of binding bulky polymer chains, could function effectively as depolymerisers, while urethanases could cleave urethane bonds in smaller fragments, enhancing overall degradation efficiency through cooperative action.

## 4 Conclusions

Our study presents PUR-GEN, a user-friendly web application designed to facilitate the generation of diverse libraries of PUR fragments. Given the heterogeneous nature of PURs and the broad substrate spectrum necessary for their biodegradation, PUR-GEN stands out as a valuable tool. Through its intuitive web-based interface, PUR-GEN facilitates the generation of a varied array of PUR compounds, empowering researchers to delve into an extensive range of PUR structures and functionalities.

An important feature of PUR-GEN is its capability to supply ready-to-use structural files and conformers for the generated oligomers. This simplifies computational studies involving PUR fragments, speeding up research processes. Furthermore, the tool allows direct access to the properties of generated ligands, enabling efficient comparison and characterisation of PUR compound libraries.

In a use case study presented in this work, PUR-GEN was applied to prepare a library comprising 414 diverse ready-to-use PUR fragments for docking studies. Despite the size of the library, PUR-GEN enabled rapid preparation of these structures. This expedited workflow underscores the tool's scalability and efficiency for high-throughput computational investigations. By facilitating the rapid generation of ligand structures, PUR-GEN enhances researchers' capacity to identify promising enzyme scaffolds for further optimisation, thus contributing to the advancement of enzymatic solutions for PUR degradation.

The presented case study shows that valuable results can be obtained even with simple approach (AutoDock Vina [49], [50] employs a rigid docking protocol, and the conformations of proteins used in this study may be not optimal). Methods based on flexible docking, or molecular dynamics to explore more carefully conformational space of targeted proteins could serve as an extension of proposed pipelines aiming preselection of protein candidates for future engineering. Regardless of whether sophisticated methods are used to analyse the degradation potential of specific enzymes, the proposed webserver, PUR-GEN, simplifies and facilitates the construction of a library of PUR fragments for such studies. As substrate binding is the first step of the reaction, the prepared library of PUR fragments was used in a molecular docking study which can serve to select enzyme folds for further redesign to produce highly efficient PUR-degrading enzymes and to design PUR degradation pipelines. While our study focused on a specific application, the versatility of PUR-GEN extends to numerous potential research domains aimed at addressing the environmental challenges posed by PUR waste management.

## 5 Methods

### 5.1 Ligands preparation

We used PUR-GEN tool to generate PUR fragments from selected structural units: 10 isocyanates (Supplementary Figure S1.) and 16 alcohols (Supplementary Figure S2.). PUR structures consisted of 2, 3, and 4 structural units, resulting in a total of 414 compounds. Free isocyanate moieties in obtained fragments were replaced by amine (-NH2) groups. Structures were further converted from the.mol2 to the.pdbqt format by using script provided in AutoDock Vina software [49], [50], “prepare_ligand4.py”. Additionally, we used “-U \'\'” option to keep the structures in their protonated form.

Properties of the generated ligands — including molecular weight, heavy atom count, number of rotatable bonds, presence of ester bonds, presence of ether bonds, count of aromatic atoms, aromatic proportion (ratio of aromatic atoms to heavy atoms), Crippen-Wildman partition coefficient (cLogP), Crippen-Wildman molar refraction (MR) [46], and topological polar surface area (TPSA) [47] — were also downloaded from the PUR-GEN website.

### 5.2 Proteins preparation

Crystal structures of selected bacteria cutinases from *Thermobifida fusca* (TfCut2, Protein Data [75] Bank (PDB) id: 4CG1 [51]) and leaf compost metagenome (LCC, PDB id: 4EB0 [76], fungal cutinase from *Humicola insolens* (HiC, PDB id: 4OYY [52]), were retrieved in.pdb format from Protein Data Bank. Crystallographic water and ions were removed from these structures. The structures of fungal cutinase from *Cladosporium sp.* P7 (CpCut1) and selected urethanases: UMG-SP-1, UMG-SP-2 and urethanase from *Agrobacterium tumefaciens (*AmdA) were modelled using AlphaFold 2 [77] based on provided sequence (GenBank: OR245267, WBR49956, WBR49957, AAK28498, respectively). Although a recent crystal structure of the urethanase UMG-SP-1 is available (PDB id: 8S7Z [61]), it contains unresolved regions, including a missing loop with the catalytic residue S152. Therefore, we opted to use an AlphaFold 2 model. Proteins were protonated with the use of H++ server [78] at pH 8.0 for TfCut2, LCC, HiC and UMG-SP-2, and at pH 7.5 for UMG-SP-1 and AmdA. Protonated structures were converted to.pdbqt format with the “preparereceptor4.py” script provided with AutoDock Vina software. Structural multiple sequence alignments (MSA) of the four cutinases and three urethanases were prepared using MUSTANG [79] and visualised using EsPript [80].

### 5.3 Docking

Molecular docking with rigid receptors and flexible ligands was performed using AutoDock Vina v1.2.3 [49], [50]. The docking region was defined around the catalytic residues in the binding pockets of the receptors, specifically targeting the catalytic serine residues: S130 for TfCut2, S165 for LCC, S105 for HiC, S129 for CpCut1, S176 for UMG-SP-1, S175 for UMG-SP-2 and S197 for AmdA. The dimensions of the docking boxes were set to 30.00 × 37.50 × 22.50 Å for TfCut2 and LCC, 30.00 × 26.25 × 26.25 Å for HiC and CpCut1, and 33.75 × 37.50 × 33.75 Å for UMG-SP-1, UMG-SP-2 and AmdA. Each docking simulation was configured to generate 10 ligand poses and was repeated 10 times, yielding approximately 100 poses per ligand. The positioning of the docked ligands and their binding affinities, as indicated by the Vina score, were analysed. Ligand poses with a hydrolysable bond (urethane or ester) located within 4.5 Å of the catalytic serine and oxyanion residues were classified as productive poses. For oxyanion stabilisation, backbone hydrogens from residues Tyr60 and Met131 for TfCut2, Ser28 and Glu106 for HiC, Tyr95 and Met166 for LCC, Ser50 and Glu130 for CpCut1, Ile173 and Gly174 for UMG-SP-1, Ile172 and Gly173 for UMG-SP-2, and Gln194 and Gly195 for AmdA were considered. Distance calculations and analysis were performed using Python with the Biopython package [81]. Data handling and processing were carried out using Pandas [82] and NumPy [83]. Plots and visualisations of the results were generated using Matplotlib [84] and Seaborn [85]. Visualisation of the docking results was performed using PyMOL 2.5.7 [86].

## CRediT authorship contribution statement

**Katarzyna Szleper:** Data curation, Formal analysis, Investigation, Methodology, Software, Visualisation, Roles/Writing – original draft, Writing – review & editing. **Mateusz Cebula:** Software, Validation. **Oksana Kovalenko:** Validation, Writing – review & editing. **Artur Góra:** Conceptualisation, Supervision, Writing – review & editing. **Agata Raczyńska:** Data curation, Formal analysis, Funding acquisition, Project administration, Validation, Visualisation, Roles/Writing – original draft, Writing – review & editing.

## Declaration of Generative AI and AI-assisted technologies in the writing process

During the preparation of this work the authors used ChatGPT to improve readability and language. After using this tool, the authors reviewed and edited the content as needed and take full responsibility for the content of the publication.

## Declaration of Competing Interest

The authors declare that they have no known competing financial interests or personal relationships that could have appeared to influence the work reported in this paper.

## Data Availability

Generated data is available in the Zenodo repository (https://doi.org/10.5281/zenodo.11612378). The source code for the PUR-GEN website is openly accessible on GitHub (https://github.com/kataszl203/pur-gen).
