# Supplementary material for: PUR-GEN: A web server for automated generation of polyurethane fragment libraries
Source: Comput Struct Biotechnol J. 2024 Dec 12;27:127–36. doi: 10.1016/j.csbj.2024.12.004 (PMC11750484; doi:10.1016/j.csbj.2024.12.004)
Supplement: Supplementary file 1 — Supplementary material [file mmc1.docx]

# Supplementary Materials

# PUR-GEN: A Web Server for Automated Generation of Polyurethane Fragment Libraries

**Katarzyna Szleper**, Mateusz Cebula, Oksana Kovalenko, Artur Góra*,
Agata Raczyńska*

**
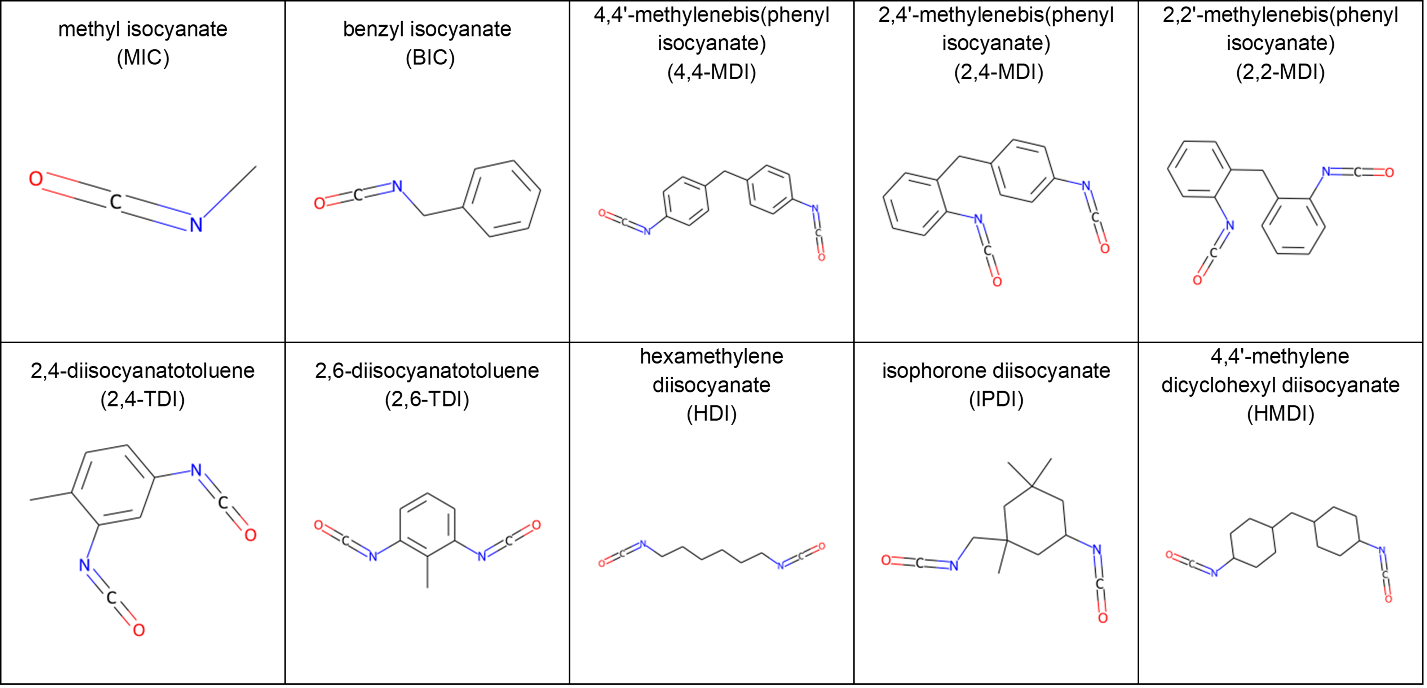
**

**Figure S1.** Isocyanates available in PUR-GEN and selected for PUR generation in the case study presented.


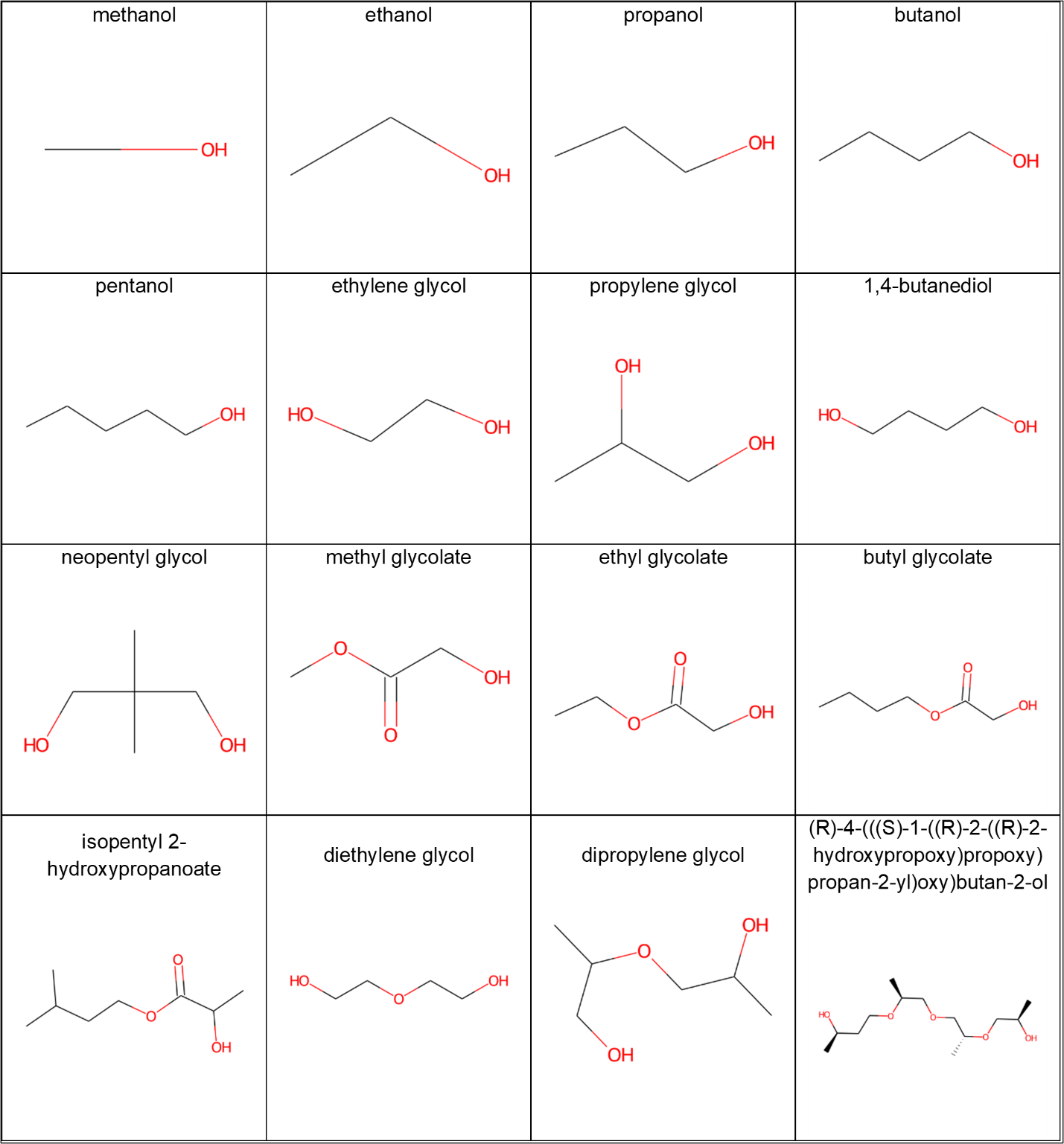


**Figure S2.** Alcohols available in PUR-GEN and selected for PUR generation in the case study presented.


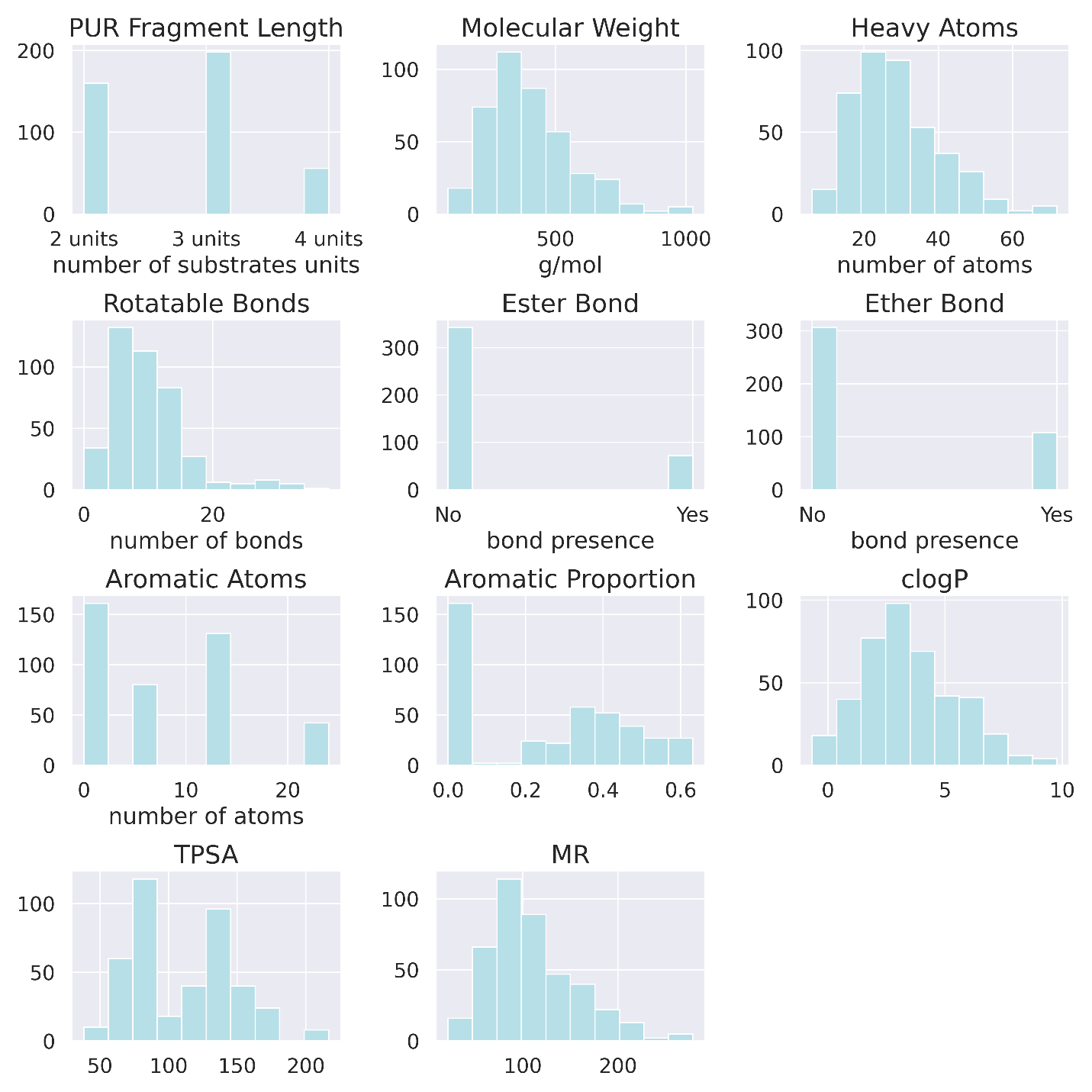


**Figure S3.** Distribution of various properties in the generated PUR library using PUR-GEN: **a)** PUR fragment length, **b)** molecular weight, **c)** heavy atom count, **d)** number of rotatable bonds, **e)** presence of ester bonds, **f)** presence of ether bonds, g) count of aromatic atoms, **h)** aromatic proportion (ratio of aromatic atoms to heavy atoms), **i)** Crippen-Wildman partition coefficient (cLogP), **k)** topological polar surface area (TPSA), **j)** Crippen-Wildman molar refraction (MR).


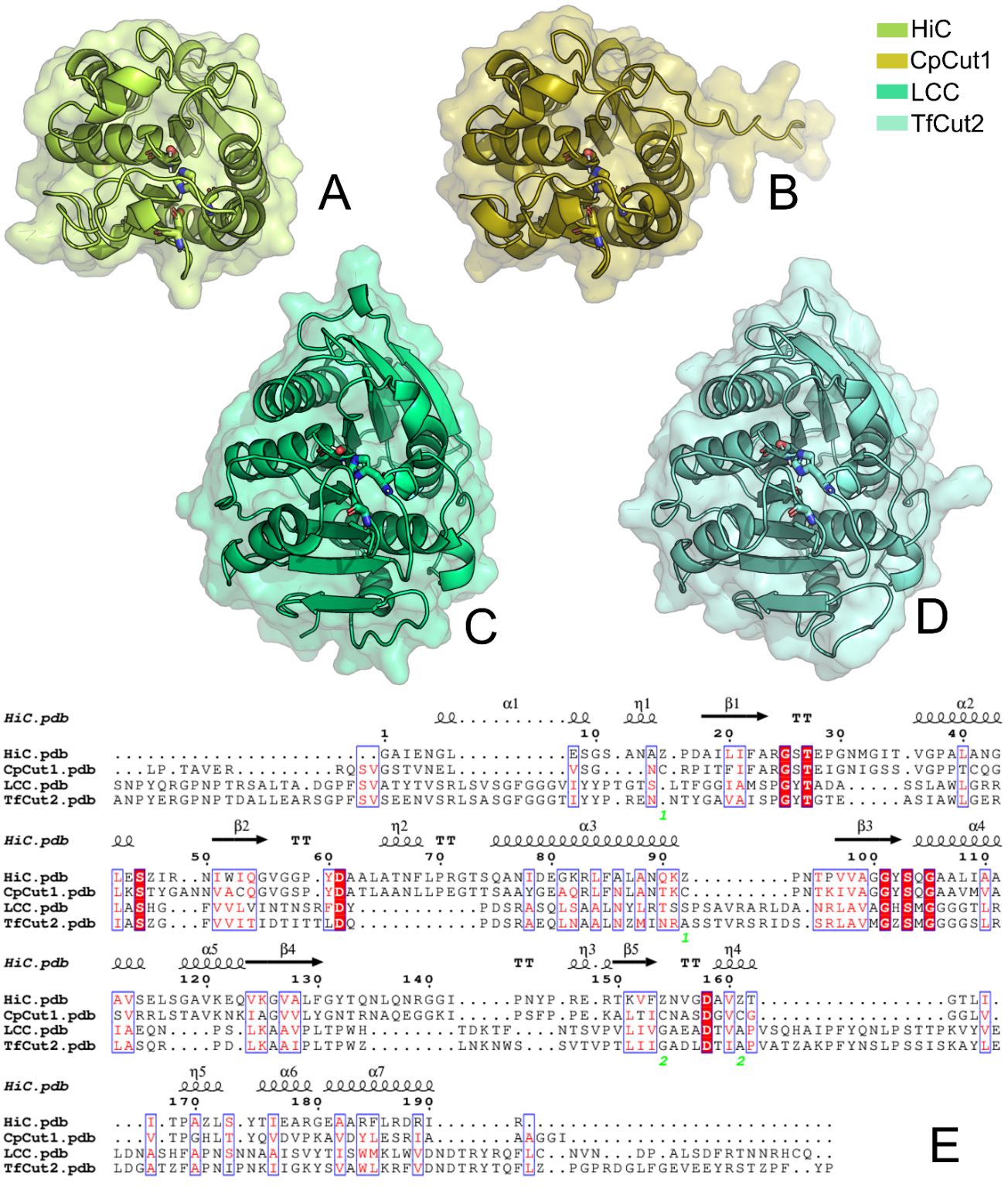


**Figure S4.** Aligned structures and structural MSA of four cutinases. Structures of cutinases are shown as cartoons with catalytic triad Ser-His-Asp residues displayed as sticks. The protein surface is visible in the background. **A)** HiC; **B)** CpCut1; **C)** LCC; **D)** TfCut2; **E)** MSA of the four cutinases: HiC, CpCut1, LCC, and TfCut2. The secondary structure of HiC, based on its crystallographic structure deposited in PDB (PDB id: 4OYY), is indicated above the aligned sequences. The structural MSA was prepared using MUSTANG and visualised in EsPript.


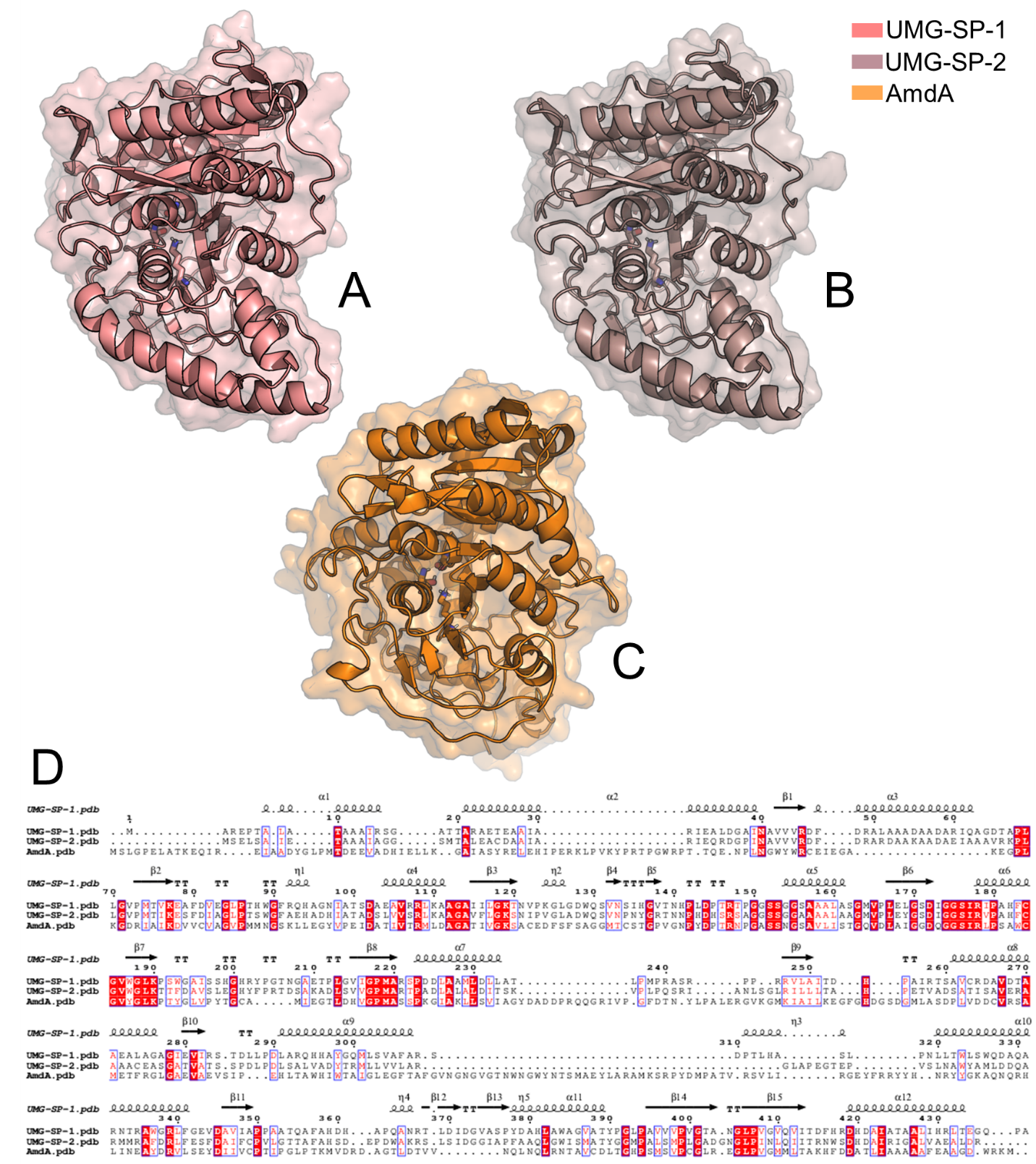


**Figure S5.** Aligned structures and structural MSA of three urethanases. Structures of urethanases are shown as cartoons with catalytic triad Ser-(cis)Ser-Lys residues displayed as sticks. The protein surface is visible in the background. **A)** UMG-SP-1; **B)** UMG-SP-2; **C)** AmdA; **D)** MSA of the three urethanases: UMG-SP-1, UMG-SP-2 and AmdA. The secondary structure of UMG-SP-1, based on its AlphaFold 2 model, is indicated above the aligned sequences. The structural MSA was prepared using MUSTANG and visualised in EsPript.


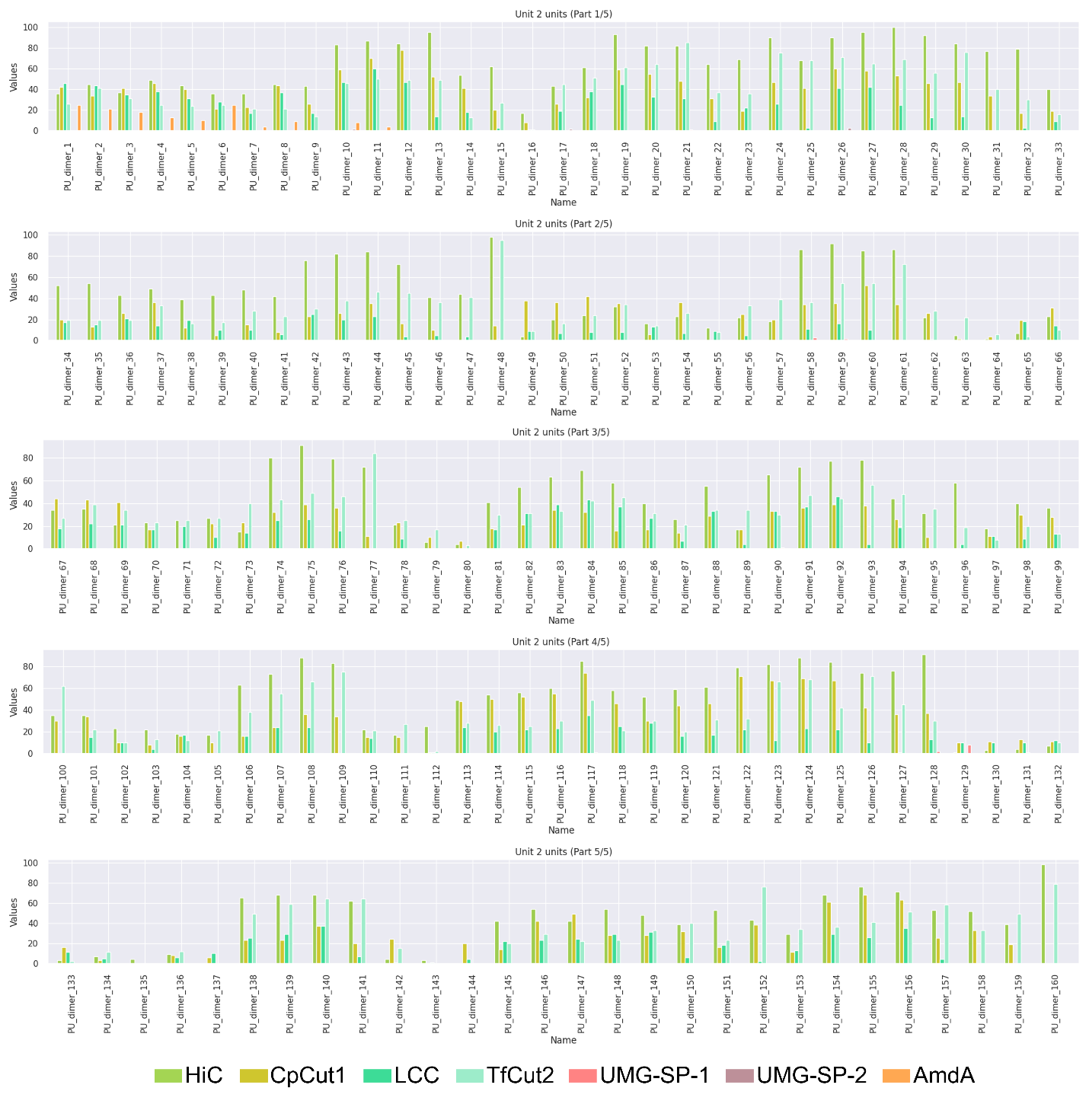
**Figure S6.** Number of productive poses obtained for PUR fragments of 2 units lengths.


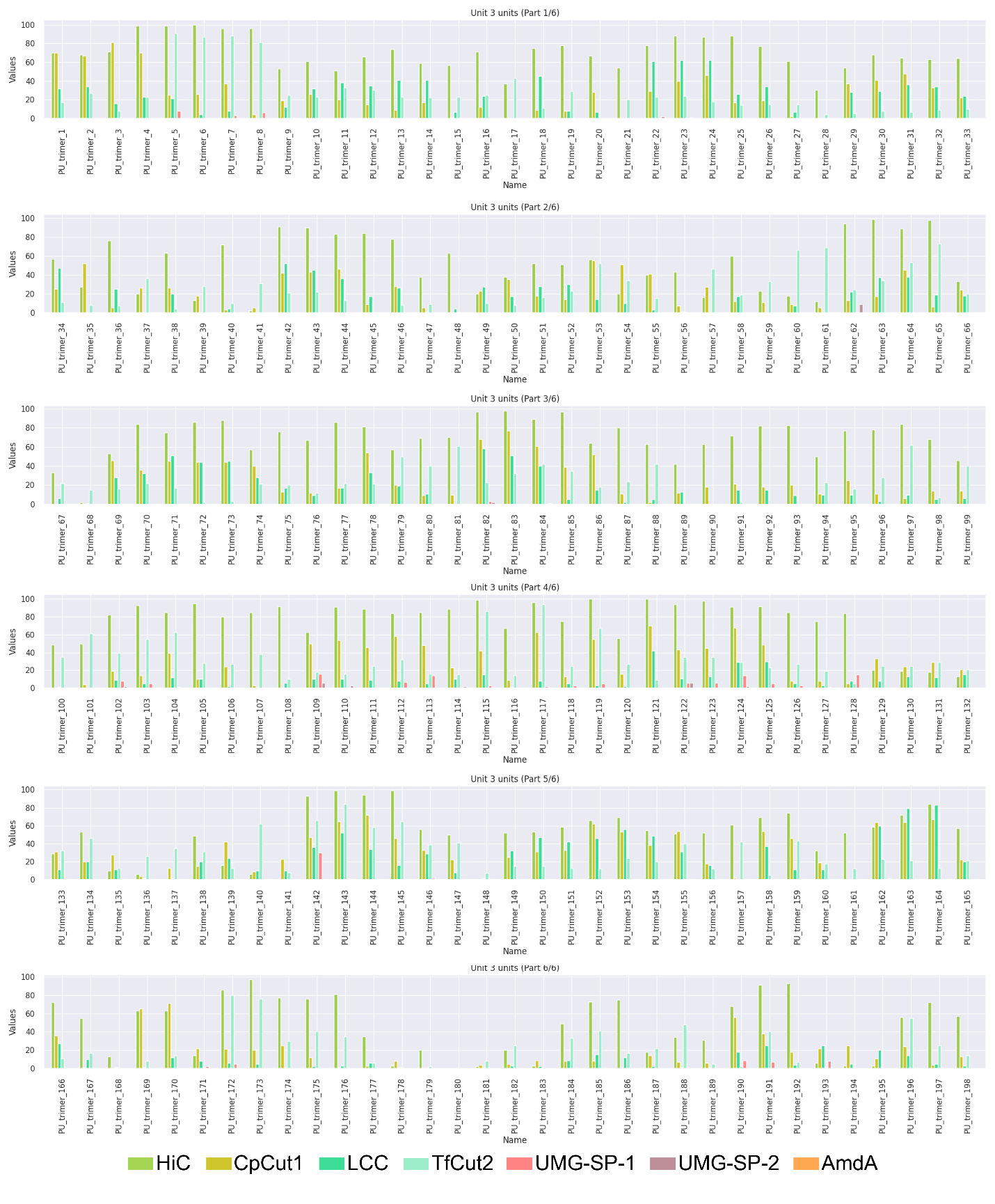
**Figure S7.** Number of productive poses obtained for PUR fragments of 3 units lengths.


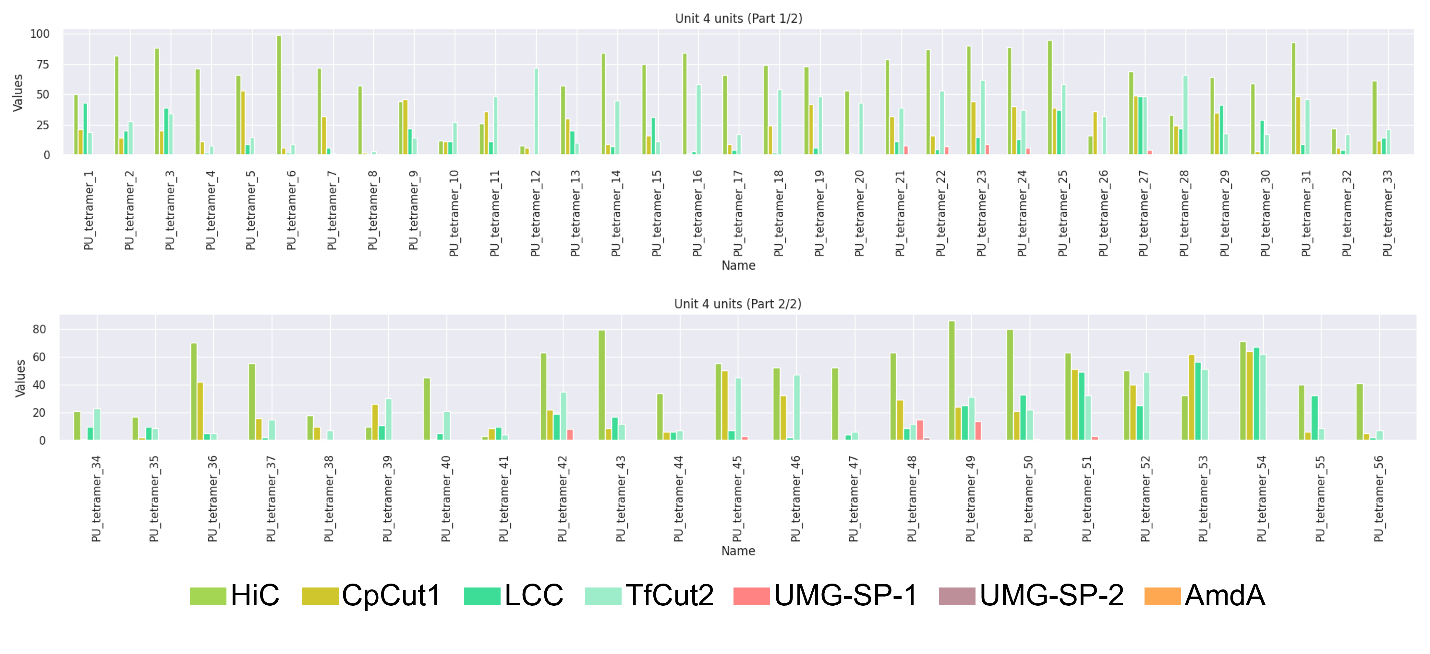
**Figure S8.** Number of productive poses obtained for PUR fragments of 4 units lengths.
